# Case report: First case report of an Emirati child with a novel gene variant causing aromatic L-amino acid decarboxylase deficiency

**DOI:** 10.3389/fped.2022.964201

**Published:** 2022-08-30

**Authors:** Mohamed O. E. Babiker, Manju A. Kurian, Jehan Suleiman

**Affiliations:** ^1^Neurosciences Centre, Al Jalila Children's Specialty Hospital, Dubai, United Arab Emirates; ^2^Developmental Neurosciences Department, University College London (UCL) Great Ormond Street Institute of Child Health, London, United Kingdom; ^3^Department of Neurology, Great Ormond Street Hospital for Children NHS Foundation Trust, London, United Kingdom; ^4^Division of Neurology, Department of Pediatrics, Tawam Hospital, Al Ain, United Arab Emirates; ^5^College of Medicine and Health Sciences, United Arab Emirates University, Al Ain, United Arab Emirates

**Keywords:** AADC deficiency, case report, oculogyric crises, dystonia, hypotonia, delayed diagnosis, developmental delay, UAE

## Abstract

Aromatic L-amino acid decarboxylase (AADC) deficiency is a rare, neurometabolic disorder resulting from biallelic mutations in the dopa decarboxylase *(DDC)* gene. This is the first reported case of AADC deficiency in the United Arab Emirates (UAE) and describes an Emirati male patient who presented in the first few months of life with a severe phenotype of global hypotonia, developmental delay and oculogyric crisis. Following whole exome sequencing, a novel homozygous mutation in the *DDC* gene (c.1144G>T, p.Val382Phe) was reported and the patient underwent further testing, after which a diagnosis of AADC deficiency was confirmed. This mutation has not been previously described, but the clinical phenotype and corresponding biochemical profile confirmed that it is a pathogenic variant. The patient is currently managed at a tertiary referral center in the UAE and is treated in accordance with published guidance on AADC deficiency, including the recommended medical therapy combined with multidisciplinary care from a team of specialists. Some symptomatic improvements have been reported but at 5 years of age the patient continues to exhibit profound developmental delay, oculogyric crisis and is prone to recurrent respiratory infections. In order to improve outcomes for patients with AADC deficiency in the Middle Eastern region, there is an urgent need to raise the index of clinical suspicion, particularly among primary care physicians, pediatricians, and pediatric neurologists, and to improve access to diagnostic testing. This is particularly relevant at the current time, given the ongoing development of potentially disease-modifying gene therapy for AADC deficiency.

## Introduction

Aromatic L-amino acid decarboxylase (AADC) deficiency is a rare, autosomal recessive neurotransmitter disorder resulting from mutations within the dopa decarboxylase (*DDC*) gene (1). Since it was first reported in 1990 (1), at least 135 patients with AADC deficiency have been described in the medical literature (2) and 260 separate mutations are currently listed in the BioPKU database (http://www.biopku.org/home/biopku.asp). Although the true global prevalence of AADC deficiency is unknown (3), an increased prevalence has been reported in Asian populations, possibly due to a founder mutation (4).

The AADC enzyme is required for the final step in the synthesis pathway of monoamine neurotransmitters; it catalyzes the conversion of L-DOPA and 5-hydroxytryptophan to dopamine and serotonin, respectively (5). Dopamine functions as both a neurotransmitter and a precursor for the hormones noradrenaline and adrenaline (5). As a result, AADC deficiency leads to a combined depletion of dopamine, serotonin, noradrenaline, and adrenaline (1), resulting in severe neurological manifestations, developmental disabilities, and risk of early death (3, 6, 7). Although clinical phenotypes can vary widely, the signs and symptoms most commonly associated with AADC deficiency include hypotonia and motor impairment, oculogyric crises, autonomic dysfunction, feeding difficulties, failure to thrive, and delayed development (3, 8, 9). Symptom onset is typically within the first few months of life for patients with recessive loss-of-function mutations in the *DDC* gene. However, diagnosis of AADC deficiency is frequently delayed, with an average age at diagnosis reported to be 3.5 years (3). This is partly due to the symptoms of AADC deficiency being non-specific, resulting in patients being misdiagnosed with more prevalent conditions such as cerebral palsy and epilepsy (10). Diagnostic delays can be further exacerbated by a lack of disease awareness among community physicians (11).

Consensus guidelines on the diagnosis of AADC deficiency recommend genetic testing in addition to at least one other functional test such as the biochemical evaluation of the levels of neurotransmitter metabolites in cerebrospinal fluid (CSF) or analysis of plasma AADC activity (3). There are three key diagnostic tests for identifying AADC deficiency: (i) compound heterozygous or homozygous pathogenic *DDC* gene variants; (ii) low CSF levels of 5-hydroxyindoleacetic acid, homo-vanillic acid, and 3-methoxy-4-hydroxyphenylglycol, normal CSF pterins, and high CSF levels of 3-O-methyldopa, L-DOPA, and 5-hydroxytryptophan; and (iii) decreased plasma AADC enzyme activity (3).

Currently, first-line medications for AADC deficiency include dopamine agonists in combination with monoamine oxidase inhibitors and pyridoxine; additional medications can also be used for control of symptoms, such as anticholinergics and melatonin (3). However, for most patients, these treatments offer limited benefit (3, 12). In addition to medical treatments, a multidisciplinary approach is recommended to prevent secondary complications and promote development (3). This includes physiotherapy, speech therapy, occupational therapy, feeding and nutritional assessment, and neuropsychological treatment (3). An attractive area for future research is gene therapy, with recent studies demonstrating clinical improvements following the addition of a functional *DDC* gene to the basal ganglia using an adeno-associated viral vector to primarily target dopaminergic systems (13–16). In these studies, younger age has been associated with improved treatment outcomes; therefore, there is a clear need for early diagnosis (14, 15).

**Figure 1:**
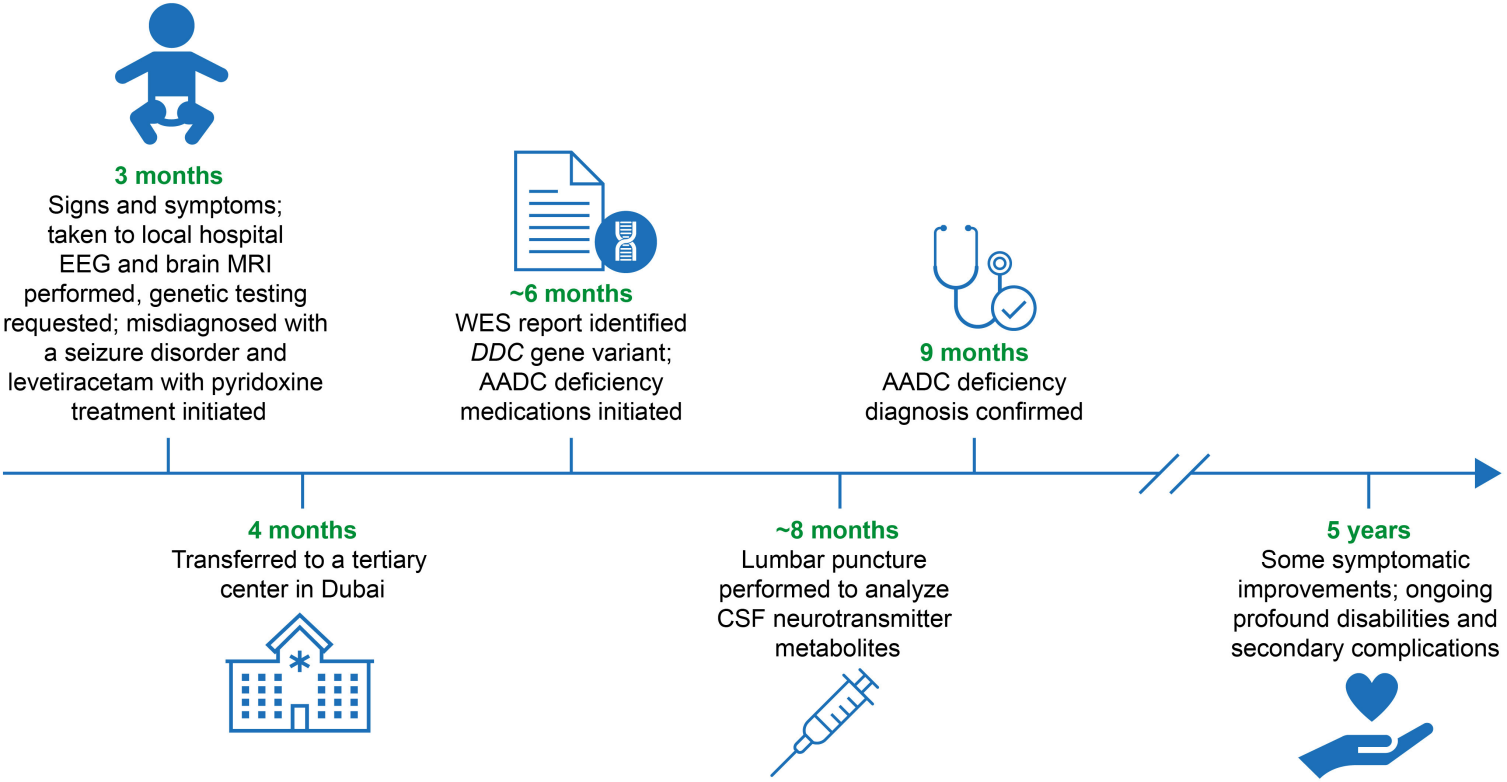
Diagnostic and patient management timeline. AADC, aromatic L-amino acid decarboxylase; CSF, cerebrospinal fluid; *DDC*, L-DOPA decarboxylase; EEG, electroencephalogram; MRI, magnetic resonance imaging; WES, whole exome sequencing.

Improvements to the accurate and timely diagnosis of AADC deficiency around the world, including in the Middle East, are crucial to ensure patients achieve early access to multidisciplinary management teams and potentially life-changing treatments. The objective of this case report is to examine the clinical signs and symptoms, diagnosis, and management of a confirmed case of AADC deficiency from the United Arab Emirates (UAE) with a novel *DDC* gene variant (Figure 1).

## Case presentation

This case study is of an Emirati boy who was born at 34 weeks gestation by emergency lower segment Cesarean section owing to fetal bradycardia following an unremarkable pregnancy. At the time of birth, no resuscitation was required and his Apgar scores were satisfactory. The patient was the third child born to healthy, consanguineous parents. Both of the patient's older siblings had died in childhood from an undiagnosed suspected neurodegenerative disorder. For both siblings, the results from a basic metabolic panel blood test were normal; however, no genetic testing was completed and the siblings' conditions remained undiagnosed.

At the age of 3 months, the patient presented with episodes of acute, recurrent limb and body stiffening, together with recurrent, sustained upward gaze, suggestive of oculogyric crises, that could last up to 8 hours and occurred almost daily. Assessments carried out at the family's local hospital included an electroencephalogram, a brain magnetic resonance imaging scan, and blood chemistry analysis of liver function, serum lactate, and ammonia. Results of all these tests were normal. At this time, the patient was started on levetiracetam with pyridoxine treatment at therapeutic doses for suspected focal epileptic seizures. At the age of 5 months, the patient presented with another prolonged episode of recurrent limb and body stiffening. The patient was admitted to hospital and treated with an infusion of phenytoin; ongoing maintenance therapy with phenobarbitone was initiated. Despite these treatments, the patient continued to have recurrent episodes of sustained oculogyric crises that could persist for up to 4 hours. He was also troubled by recurrent periods of unsettledness, limb twisting, and stiffening, which are now believed to be dystonic attacks, and autonomic symptoms including excessive sweatiness, drooling, and nasal congestion. Recurrent respiratory infections and unexplained bouts of excessive crying and insomnia were also reported at this time. On physical examination, his growth parameters, including weight, height, and head circumference, were below the third centile for his age, and he displayed subtle dysmorphism, including prominent nasal labial area, an overhanging upper lip, a relative micrognathia, and an undescended left testicle. Generalized hypotonia was evident, with preserved deep tendon reflexes. At times, he had intermittent dystonic posturing of the neck with back arching, a mild bilateral ptosis, and extraocular movements that were intact in all directions but with an intermittent sustained upward gaze. Developmental assessment revealed global developmental delay.

Given the severe symptoms and signs, the negative investigations from brain imaging and electroencephalogram studies, the consanguinity of the parents, and the positive family history of early death in other siblings, a genetic cause was suspected. Whole exome sequencing was requested when the patient was ~4–5 months old, and identified a novel homozygous mutation in the *DDC* gene (c.1144G>T, p.Val382Phe). This missense mutation results in the substitution of a conserved valine into a phenylalanine at position 382 within the C-terminal of the protein. Both parents were subsequently shown to be heterozygous for this mutation. The variant was reported as a variant of unknown significance. Following the genetic results, a lumbar puncture was performed and CSF analysis revealed a neurotransmitter profile consistent with AADC deficiency, including reduced catecholamine metabolites, normal pterin levels, and elevation of 3-O-methyldopa (Table 1). Based on the genetic results, the CSF neurotransmitter analysis and the clinical manifestations, a diagnosis of AADC deficiency was confirmed at 9 months of age.

**Table 1:** CSF fluid neurotransmitter analysis results.

**CSF neurotransmitter**	**Patient result**	**Normal age-related reference range**
Homo-vanillic acid	48 nmol/L	176–851 nmol/L
5-hydroxyindoleacetic acid	13 nmol/L	68–451 nmol/L
Neopterin	11 nmol/L	7–65 nmol/L
Dihydrobiopterin	12 nmol/L	0.4–13.9 nmol/L
Tetrahydrobiopterin	43 nmol/L	19–56 nmol/L
5-methyltetrahydrofolate	113 nmol/L	72–305 nmol/L
3-O-methyldopa	1,543 nmol/L	<100 nmol/L
CSF pyridoxal phosphate	79 nmol/L	14–92 nmol/L
CSF glucose	2.9 mmol/L	2.8–4.4 mmol/L
CSF lactate	1.3 mmol/L	1.2–2.1 mmol/L

Abnormal findings/values (outside of reference ranges provided for the assays used) are indicated in red.

CSF, cerebrospinal fluid.

Following the whole exome sequencing report at ~6 months of age, anti-seizure medications were withdrawn and the patient was instead treated with medications recommended by the guidelines on the diagnosis and treatment of AADC deficiency (3), including pyridoxine, selegiline (monoamine oxidase inhibitor), bromocriptine (dopamine agonist), and trihexyphenidyl (anticholinergic), in addition to calcium folinate, melatonin, clonidine, and chloral hydrate, to help relieve autonomic symptoms, sleep disturbance, and irritability.

Trihexyphenidyl treatment was subsequently discontinued owing to excessive sleeping and constipation, and clonidine was discontinued owing to excessive sedation. Echocardiograms performed to screen for bromocriptine-associated cardiac fibrosis or valvopathy were performed according to the treatment guidelines and have so far been normal. Following the change in medication, the frequency of oculogyric crises reduced from almost daily to weekly or fortnightly episodes. Furthermore, some improvements were noted in other symptoms, including sleep and control of limb twisting.

As of September 2021, the patient is aged 5 years and has significantly delayed global and motor development. He is unable to sit or reach out and he has no expressive language. His receptive language is slightly more advanced. He can understand a few words, has a minimal grasp of social cues, and can smile responsively. Owing to feeding difficulties coupled with severe gastroesophageal reflux, the patient underwent a percutaneous endoscopic gastrostomy tube insertion and a Nissen fundoplication at 2 years of age. He currently receives all feeds and medications *via* the percutaneous endoscopic gastrostomy tube. Owing to functional complications of the disease, the patient has experienced recurrent respiratory infections requiring multiple hospitalizations. He is currently being monitored every 2–4 months at a tertiary care center and his complex care needs are being managed by a specialist multidisciplinary team, comprising a pediatric neurologist, a general pediatrician, a physiotherapist, a pulmonologist, a gastroenterologist, a dietician, and a cardiologist. Genetic counseling has also been offered to the patient's family.

## Discussion

In this case report we describe a patient from the UAE with AADC deficiency caused by a novel homozygous variant within the *DDC* gene (c.1144G>T) resulting in a single amino acid substitution (Val382Phe) within the C-terminal domain of the AADC enzyme (5). The previously reported pathogenic mutations span the length of the *DDC* gene, with variants in a similar location (exon 13) outlined in previous case reports (9, 17). The mutation was classified as a variant of unknown significance as it has not been previously described and its impact on the structure and function of the enzyme is unknown; however, the severe and clinically compatible phenotype and biochemical data support variant pathogenicity. This is the first detailed case report of AADC deficiency in the UAE and is amongst very few case reports in the Middle Eastern region (2, 18). However, owing to consanguineous marriages being relatively common in the Middle Eastern region for cultural and socioeconomic reasons (19, 20), there is an increased risk of autosomal recessive disorders (21) and it is therefore likely that AADC deficiency may be more prevalent than is currently reported in the UAE.

The patient in this case presented with a severe phenotype shortly after birth, with signs and symptoms consistent with other case reports (2, 17). Diagnostic delays are common in patients with AADC deficiency owing to many symptoms being non-specific combined with primary care physicians having a low index of suspicion for the disease (2). In this case, the slight delay in diagnosis was largely due to the non-specific presentation of the disease, with oculogyric crises being mistaken for focal seizures. However, based on the known parental consanguinity and the earlier sibling history, genetic testing was performed relatively early for this patient (at ~6 months of age) and therefore the diagnostic delay was not as pronounced as has been reported for many other patients.

Since the age of 6 months, the patient has been treated with a combination of recommended medications. These have been generally well tolerated and some improvements in symptoms have been noted, particularly the frequency of oculogyric crises and control of dystonia. However, the patient continues to have a profound global developmental delay and ongoing secondary complications.

A strength of this study is that it highlights the diagnostic challenges faced by healthcare professionals in the UAE who are managing patients with this rare condition. Limitations of this study are that it is based on a single patient case and, as for all case reports, it is vulnerable to selection and recall bias.

Early diagnosis of AADC deficiency is critical for enabling the provision of early access to targeted treatments for patients, multidisciplinary management, and for avoiding treatment with unnecessary and potentially harmful medications. Accurate genetic diagnosis of autosomal recessive disorders has many important implications for families of affected individuals, particularly for further family planning to help prevent recurrence, with *in vitro* fertilization and preimplantation genetic testing now available choices for these families.

Once gene therapy is available, early diagnosis will be particularly crucial to ensure treatment is provided promptly to treat clinical symptoms and promote neurodevelopment. The European Commission has recently granted marketing authorization for PTC Therapeutics' product Upstaza^TM^ (eladocagene exuparvovec), an *in vivo* gene therapy for treating AADC deficiency through delivery of the *DDC* gene to the putamen. It is approved for patients aged 18 months of age and older (22). A clinical trial of another gene therapy approach for AADC deficiency, targeting the midbrain, has successfully met its safety and primary and secondary efficacy endpoints (13). Raising the index of suspicion for the disease, particularly amongst primary care physicians and pediatric neurologists, along with providing earlier access to genetic testing, will be key to ensuring that patients are rapidly diagnosed following initial symptom presentation. Additionally, the introduction of screening programs utilizing non-invasive diagnostic procedures at all primary care centers, such as the analysis of plasma AADC activity or the use of dried blood spot sampling to measure 3-O-methyldopa (23), when a child presents with developmental delay and/or hypotonia could aid early diagnosis. However, such early screening would need to be validated with molecular genetic investigations for confirmatory diagnosis (3).

To date, few cases of AADC deficiency have been described in the Middle East, although the number of cases is expected to rise given the increasing availability of whole exome sequencing genetic tests. Gene therapy holds great promise for children affected by this disease; however, additional challenges related to the availability and affordability of gene therapy in Middle Eastern countries may arise in the future.

## Data availability statement

The datasets for this article are not publicly available due to concerns regarding participant/patient anonymity. Requests to access the datasets should be directed to the corresponding author.

## Ethics statement

Written informed consent was obtained from the minor(s)' legal guardian/next of kin for the publication of any potentially identifiable images or data included in this article.

## Author contributions

MB and JS were involved in the conception of the paper. All authors were involved in the writing and reviewing of the manuscript and approved the final draft.

## Funding

Medical writing and editorial support were funded by PTC Therapeutics. PTC Therapeutics had no involvement in the writing of the report.

## Conflict of interest

Authors MB and JS have received an honorarium from PTC Therapeutics for advisory work. The remaining author declares that the research was conducted in the absence of any commercial or financial relationships that could be construed as a potential conflict of interest.

## Publisher's note

All claims expressed in this article are solely those of the authors and do not necessarily represent those of their affiliated organizations, or those of the publisher, the editors and the reviewers. Any product that may be evaluated in this article, or claim that may be made by its manufacturer, is not guaranteed or endorsed by the publisher.
